# A Medical Student Initiative to Enhance the Pediatric Hemodialysis Experience

**DOI:** 10.3928/24748307-20210126-01

**Published:** 2021-03-08

**Authors:** Jennifer Ferrante, Stephanie S. Camhi, Olivia Neumann, Jayanthi Chandar

## Abstract

**Background:**

Children and young adults receiving hemodialysis (HD) face unique challenges including frequent school absenteeism, psychosocial issues, and social isolation, placing them at risk for decreased academic achievement and health literacy.

**Objective:**

To address this, we implemented the Child and Adolescent Motivation and Enrichment Program (CHAMP) at Holtz Children's Hospital in Miami, FL. The objective of this study is to describe the organizational structure and program design of CHAMP and provide preliminary program opinions.

**Methods:**

Medical students served as longitudinal one-on-one mentors to patients receiving HD. Face-to-face intervention, books, board games, and electronic tablets were used to enhance patients' educational and recreational experience. We surveyed participating patients, medical students, and unit nurses regarding their opinions of CHAMP.

**Key Results:**

Patients responded to a series of questions on a Likert scale scored from 1 to 5 and reported the highest scores on questions pertaining to having fun with mentors (mean = 4.88), enjoying mentor visits (mean = 4.78), and learning during visits (mean = 3.88). Mentors reported the highest level of agreement (mean = 4.82) that CHAMP helped them gain empathy for patients with chronic and/or special health care needs. Nurses scored highly on the point that “overall, the program was useful and helped the patient” (mean = 6.86 of a possible 7).

**Conclusion:**

CHAMP is an academic and psychosocial enrichment program for children and adolescents receiving HD. The program is regarded highly by participating patients, medical students, and unit nurses. Patients report enjoying and learning from mentor sessions, whereas nurses report improved interactions with patients. Medical students who participate as mentors also gain important exposure to the field of pediatric nephrology. The program design as described herein positions CHAMP for replication at academic medical centers nationwide, allowing for optimization of the health and well-being of the pediatric HD population. **[*HLRP: Health Literacy Research and Practice*. 2021;5(1):e60–e69.]**

**Plain Language Summary:**

Pediatric patients undergoing hemodialysis (HD) are at risk for decreased academic achievement and health literacy. To address this, we implemented the Child and Adolescent Motivation and Enrichment Program, a longitudinal mentorship program pairing medical students as one-on-one mentors to patients undergoing HD. Preliminary results from this program demonstrate satisfaction and enjoyment by participating patients, medical students, and dialysis unit nurses.

Children and adolescents with end-stage kidney disease (ESKD) require renal replacement therapy (RRT) in the form of dialysis or kidney transplantation (KT) to survive. Although KT provides better quality of life than dialysis, many children require a period of dialysis prior to KT. Peritoneal dialysis is the preferred mode of RRT for children as it is more physiologic and less disruptive to a child's schooling (Zaritsky & Warady, 2011). However, medical, surgical, and psychosocial factors may compel the decision to choose hemodialysis (HD) (Collins et al., 2012). The large time commitment associated with attending HD sessions is particularly detrimental for school-aged children due to risk for decreased educational attainment and predisposition to social isolation.

ESKD, compared to other chronic illnesses, uniquely results in impaired cognitive development (Moser et al., 2013). Children who receive dialysis show decreased abilities in reading, arithmetic, and spelling compared to those who receive KT (Chen et al., 2018), with poorer cognitive outcomes specifically for patients on HD versus patients on peritoneal dialysis (Ozcan et al., 2015). Achieving full educational potential is vital to children with ESKD given the importance of health literacy in understanding one's condition and making informed health care decisions. Studies of children on dialysis have identified five themes: (1) loss of control, (2) restricted life, (3) coping strategies, (4) managing treatment, and (5) feeling different (Tjaden et al., 2012). Reduced healthy literacy and health-related quality of life, in addition to depressive symptoms, are observed in children with ESKD compared to healthy populations (Ozcan et al., 2015; Simoni et al., 1997; Varni et al., 2007).

Although the metabolic effects of chronic kidney disease (CKD) are amenable only to medical therapy, the educational and social aspects of CKD in HD patients present a unique opportunity for intervention. There exists an undeniable connection between health and education for optimal childhood development (Bundy et al., 2017). Although a wealth of research exists pertaining to early childhood development, much less focus has historically been placed upon optimizing development for children between ages 5 and 14 years (Bundy et al., 2017). Previous studies suggest that combining health and nutrition interventions with responsive stimulation improves not only short-term growth and cognition in childhood, but also provides lasting effects that persist into adulthood and positively affect social outcomes (Bundy et al., 2017).

Currently, there is a physician shortage in the field of pediatric nephrology (Primack et al., 2015). Early clinical exposure may motivate medical students to join the field. Prior studies demonstrate that early clinical experiences are linked to increased clerkship scores and interest in the exposed specialty (Saba et al., 2015) Pre-clinical pediatric exposure provides medical students with an increased ability to communicate with pediatric patients, including those with chronic conditions (Elnicki et al., 1999), and involvement in pediatric service-learning experiences has been associated with enhanced ability to provide health literacy counseling (Milford et al., 2016).

Given that children receiving HD are socially and academically vulnerable, and the fact that there is a lack of available mentorship programs within this unique population, the Child and Adolescent Motivation and Enrichment Program (CHAMP) was implemented at Holtz Children's Hospital in 2016. As a joint initiative between medical students and pediatric nephrology faculty, the objective of CHAMP is to engage young patients receiving HD in educational and social enhancement activities with the goals of supporting patient literacy, health literacy, quality of life, and interpersonal development. Secondary benefits to participating medical students include early exposure to children with ESKD and the ability to provide counseling for and learn from this unique population.

The aim of this proof-of-concept study is to detail the program design and the benefits of participation for both HD patients and medical student mentors with the goal of disseminating our program model for implementation at additional institutions. CHAMP is a quality improvement initiative for the pediatric dialysis unit, so it does not warrant specific Institutional Review Board (IRB) approval. However, all research activities, such as those that involve assessment of patients or their medical information, are reviewed and approved by the IRB at the University of Miami and all participants are assured anonymity in compliance with the Health Insurance Portability and Accountability Act. Patients age 18 years and older provided written informed consent, whereas parental consent (with assent from children age 7 to 17 years) was obtained from all enrolled minors.

## Methods

### Context

CHAMP at Holtz Children's Hospital matches medical students one-on-one with patients from the pediatric HD unit to serve as longitudinal mentors. The unit at Holtz serves between 20 and 24 patients at a given time and is unique in that it serves a high number of young-adult patients who have yet to transition to adult care. Unit staff include a nurse manager, seven nurses, a dietician, social worker, and dialysis technician. Board games, books, and television are available to patients during treatment. CHAMP also purchased several electronic tablets to enhance the educational and recreational experience for patients. Tablets permit exposure to technology that otherwise might not be available due to socioeconomic constraints. Data from a preliminary internal survey demonstrate that 47% of our patient population does not have access to a tablet at home. CHAMP mentors sit alongside their patient mentee while they undergo dialysis.

As a student organization, CHAMP and associated activities are embedded within the clinical curriculum, permitting medical students to partake in hospital-based mentorship activities. Medical students comprise the Executive Board (E-board), which is overseen by a pediatric nephrologist, and are responsible for the majority of planning and execution of CHAMP activities. All members of the E-board serve as mentors to HD patients in addition to fulfilling their specific executive role (**Table 1**).

**Table 1:**
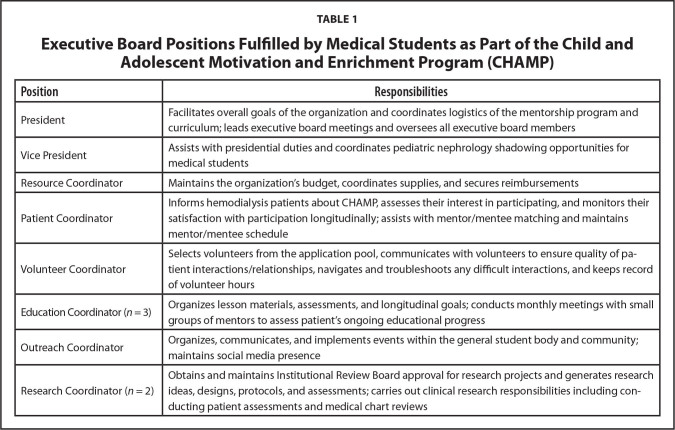
Executive Board Positions Fulfilled by Medical Students as Part of the Child and Adolescent Motivation and Enrichment Program (CHAMP)

**Position**	**Responsibilities**
President	Facilitates overall goals of the organization and coordinates logistics of the mentorship program and curriculum; leads executive board meetings and oversees all executive board members
Vice President	Assists with presidential duties and coordinates pediatric nephrology shadowing opportunities for medical students
Resource Coordinator	Maintains the organization's budget, coordinates supplies, and secures reimbursements
Patient Coordinator	Informs hemodialysis patients about CHAMP, assesses their interest in participating, and monitors their satisfaction with participation longitudinally; assists with mentor/mentee matching and maintains mentor/mentee schedule
Volunteer Coordinator	Selects volunteers from the application pool, communicates with volunteers to ensure quality of patient interactions/relationships, navigates and troubleshoots any difficult interactions, and keeps record of volunteer hours
Education Coordinator (*n* = 3)	Organizes lesson materials, assessments, and longitudinal goals; conducts monthly meetings with small groups of mentors to assess patient's ongoing educational progress
Outreach Coordinator	Organizes, communicates, and implements events within the general student body and community; maintains social media presence
Research Coordinator (*n *= 2)	Obtains and maintains Institutional Review Board approval for research projects and generates research ideas, designs, protocols, and assessments; carries out clinical research responsibilities including conducting patient assessments and medical chart reviews

### Patient Selection

All patients receiving HD on the pediatric dialysis unit at Holtz Children's Hospital are eligible to participate. An E-board member visits the unit at the beginning of each academic year to discuss the program. A brief biography is obtained from each patient including their age, educational status, goals (i.e., improved academic performance in a specific subject area, passing standardized assessments, graduating high school), and interests. Patients who originally decline to participate are reassessed periodically in case they change their mind and would like to join. Patients may elect not to participate due to access to formal tutoring during dialysis sessions or a preference to spend their time sleeping or watching television. Patients who are not actively enrolled in school are still encouraged to participate to receive companionship, mentorship, and career guidance.

### Mentor Selection, Matching, and Training

Medical students interested in becoming CHAMP mentors must complete an application and must be first-year medical students to allow for mentorship continuity throughout both years of the pre-clinical curriculum. Medical student applicants are subjected to a careful selection process and, to be selected, must demonstrate a dedication to pediatric care, previous experience working with children, and previous experience in an educational capacity. Applicants who do not have previous experience volunteering with pediatric patients are unlikely to be selected. Accepted volunteers are then matched to patient mentees considering both patient interests and biographies as well as volunteer interest and availability. After the selection process, mentors are trained regarding HD and its associated demands for the pediatric population. Training includes 2 hours of structured education (**Table 2**). Medical student mentors complete a pre-training knowledge assessment to gauge their baseline knowledge and experiences.

**Table 2:**
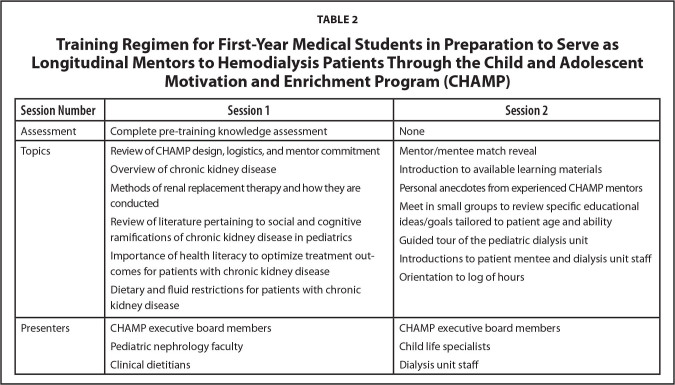
Training Regimen for First-Year Medical Students in Preparation to Serve as Longitudinal Mentors to Hemodialysis Patients Through the Child and Adolescent Motivation and Enrichment Program (CHAMP)

**Session Number**	**Session 1**	**Session 2**
Assessment	Complete pre-training knowledge assessment	None
Topics	Review of CHAMP design, logistics, and mentor commitment Overview of chronic kidney disease Methods of renal replacement therapy and how they are conducted Review of literature pertaining to social and cognitive ramifications of chronic kidney disease in pediatrics Importance of health literacy to optimize treatment outcomes for patients with chronic kidney disease Dietary and fluid restrictions for patients with chronic kidney disease	Mentor/mentee match reveal Introduction to available learning materials Personal anecdotes from experienced CHAMP mentors Meet in small groups to review specific educational ideas/goals tailored to patient age and ability Guided tour of the pediatric dialysis unit Introductions to patient mentee and dialysis unit staff Orientation to log of hours
Presenters	CHAMP executive board members Pediatric nephrology faculty Clinical dietitians	CHAMP executive board members Child life specialists Dialysis unit staff

### Program Design

**Figure 1.**
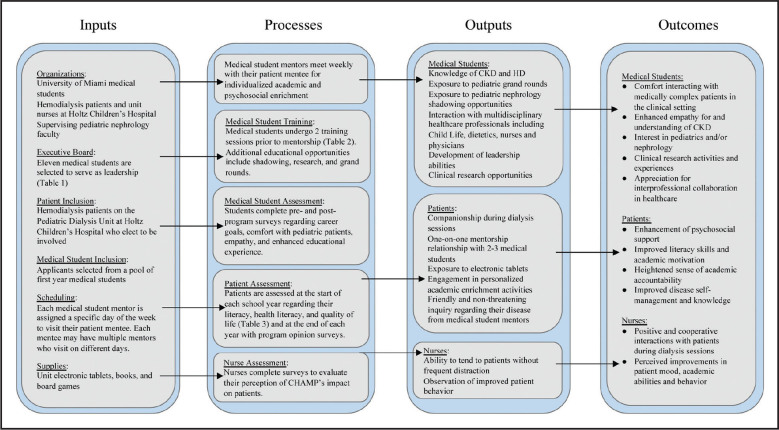
Theoretical framework depicting the inputs, processes, outputs, and outcomes associated with implementation of the Child and Adolescent Motivation and Enrichment Program (CHAMP). CKD = chronic kidney disease; HD = heart disease.

**Figure 2.**
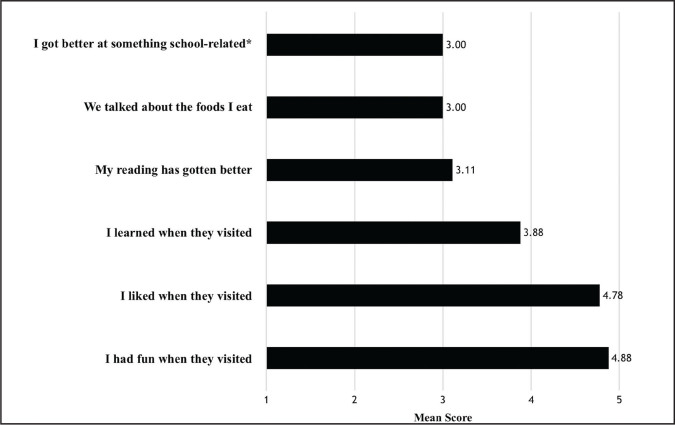
Hemodialysis patient-reported improvement in academic, disease-related, and psychosocial domains after participation in the Child and Adolescent Motivation and Enrichment Program (CHAMP). Patients participating in CHAMP report their experiences and improvements pertaining to medical-student-mentor visits to the dialysis unit. Patients responded to each question on a Likert scale ranging from 1 (*definitely no*) to 5 (*definitely yes*). Asterisk indicates other school-related improvements that refer to patients besides reading. Patients reported improvements in math, study skills, and overall academic motivation.

CHAMP mentors begin during the first year of medical school and continue once weekly interaction with their assigned patient mentee through the end of the second year (entirety of pre-clinical curriculum). The program design and outcomes are depicted in a theoretical model in **Figure 1**. HD patients electing to participate are assessed regarding their baseline literacy, health literacy, quality of life, and social support network using numerous validated questionnaires (**Table 3**). These assessments take place at the beginning of each academic year and are compared to results from the start of the previous academic year, allowing for quantification of longitudinal improvement. Patient opinions regarding program participation and their perceived educational gains are also collected using CHAMP-specific assessments at the end of each academic year (**Figure 2**).

**Table 3:**
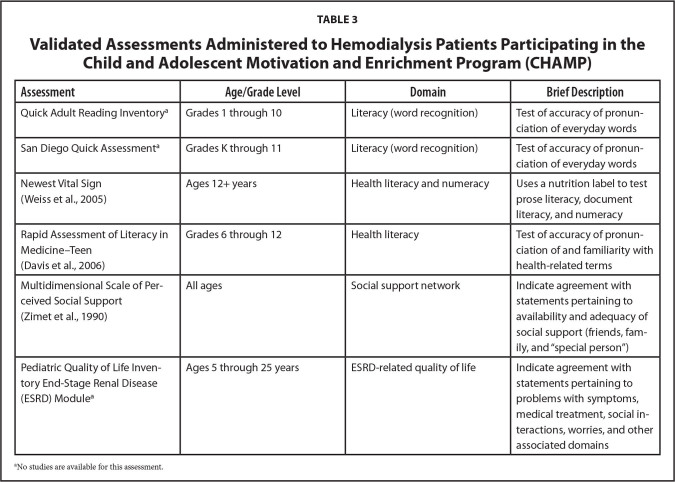
Validated Assessments Administered to Hemodialysis Patients Participating in the Child and Adolescent Motivation and Enrichment Program (CHAMP)

**Assessment**	**Age/Grade Level**	**Domain**	**Brief Description**
Quick Adult Reading Inventory^a^	Grades 1 through 10	Literacy (word recognition)	Test of accuracy of pronunciation of everyday words
San Diego Quick Assessment^a^	Grades K through 11	Literacy (word recognition)	Test of accuracy of pronunciation of everyday words
Newest Vital Sign (Weiss et al., 2005)	Ages 12+ years	Health literacy and numeracy	Uses a nutrition label to test prose literacy, document literacy, and numeracy
Rapid Assessment of Literacy in Medicine–Teen (Davis et al., 2006)	Grades 6 through 12	Health literacy	Test of accuracy of pronunciation of and familiarity with health-related terms
Multidimensional Scale of Perceived Social Support (Zimet et al., 1990)	All ages	Social support network	Indicate agreement with statements pertaining to availability and adequacy of social support (friends, family, and “special person”)
Pediatric Quality of Life Inventory End-Stage Renal Disease (ESRD) Module^a^	Ages 5 through 25 years	ESRD-related quality of life	Indicate agreement with statements pertaining to problems with symptoms, medical treatment, social interactions, worries, and other associated domains

a No studies are available for this assessment.

Each student mentor is assigned a specific day of the week to visit their mentee. A patient may have two or three medical student mentors who each visit on a different day, thus yielding a minimum of 2 to 3 hours per week of engagement with up to three distinct medical student mentors. The minimum recommended time for each interaction is 1 hour, with no maximum limit. Mentor attendance is documented through an electronic sign-out system after each visit. Mentors document total time spent with their patient mentee and indicate how much time was dedicated to educational activities. An emphasis is placed upon achieving balance between education and recreation.

Much of CHAMP's educational curriculum is individualized based on each patient's unique needs. As guidance, CHAMP provides age-specific goals addressing literacy, health literacy, social interactions, and other academic endeavors. During sessions, mentors work with their mentees to achieve both the pre-determined (CHAMP curriculum) and personal goals. For example, a “literacy” goal for younger patients may involve learning 10 new sight words over the course of 1 month, whereas a “social” goal for older patients may be creating an updated resume. Health literacy goals focus on patient understanding of potassium and phosphorus to improve disease-related knowledge. Whereas younger patients may learn to spell these words, older patients may be asked if they are aware of their levels and significance. Learning materials created by clinical dietitians at Holtz Children's Hospital specific to dietary and fluid restrictions in kidney disease are available to assist CHAMP mentors and encourage conversation surrounding these topics. Mentors frame these discussions from a perspective that encourages shared learning and adherence to their treating physician's guidelines but do not provide medical advice. Mentors do not initiate health-related discussions at every visit as these conversations may cause patients to become frustrated. Other educational activities undertaken with younger patients during mentor visits may include reading books, tracing letters, or playing counting games; older patients may receive assistance with school assignments and test preparation in addition to activities focused on career development.

### Medical Student Academic Enrichment

In addition to the core goal of enhancing the experience for pediatric and young adult patients on HD, CHAMP offers important educational enhancement opportunities for participating medical student mentors. Interaction with pediatric patients who are chronically ill allows medical students the privilege of understanding the complexities of this patient population. This experience is invaluable to physicians-in-training, and it is hoped to enhance compassion and empathy. Medical students, in addition to participating as mentors, are also able to shadow pediatric nephrologists, undertake or attend speaking engagements, and participate in clinical research activities.

### Program Opinion Surveys

After 1 year of mentorship activities, participating patients completed surveys regarding their experiences and opinions of CHAMP. Patient surveys focused on patient-perceived educational gains, improvement in disease-related knowledge, and enjoyment of mentor visits. Patients indicated their responses on a 5-point Likert scale with the anchors of *definitely no* (1), *neutral* (3), and *definitely yes* (5) with corresponding smile face images. Medical students also completed surveys including a pre-training knowledge assessment and 6-month follow-up survey. Students indicated their responses on a 5-point Likert scale with the anchors of *strongly disagree* (1), *neutral* (3), and *strongly agree* (5) to questions pertaining to career goals, comfort with pediatric patients, ability to counsel patients, empathy, understanding social determinants of health, and enhancement of their educational experience. Unit nurses also completed anonymous surveys to gauge how they perceived CHAMP to influence their patient's neurocognitive development, anxiety, and behavior. Each nurse completed a single survey to reflect opinions of their patients overall. Nurse surveys were scored on a 7-point Likert scale with the anchors of *strongly disagree* (1), *neutral* (4), and *strongly agree* (7). Nurse surveys additionally included three open-ended questions regarding other benefits of participating in the program as well as the best and worst parts about the program.

## Results

### Patient Opinions

Nine patients age 8 to 25 years (mean = 17.6 ± 6.9 years, 67% female, 22% Hispanic, 78% African American) were evaluated among 12 that participated in CHAMP during the 2018 to 2019 academic year. Patients that were not evaluated received transplants (*n* = 2) or were feeling unwell at the time of survey (*n* = 1). Results from patient surveys are depicted in **Figure 2**. Although patients were neutral to the statement “I got better at something school related [apart from reading],” (mean = 3), they reported slightly improved reading ability (mean = 3.11) and learning when mentors visited (mean = 3.88). Patients reported the highest scores on questions pertaining to having fun with mentors (mean = 4.88) and enjoying mentor visits (mean = 4.78). To a free response question addressing other academic improvements, patients reported that the program helped them “play games, change their school schedule, be more motivated, gain new perspective, get ready for college, and think about what [they] want to do/study.”

### Medical Student Opinions and Academic Enrichment

**Figure 3.**
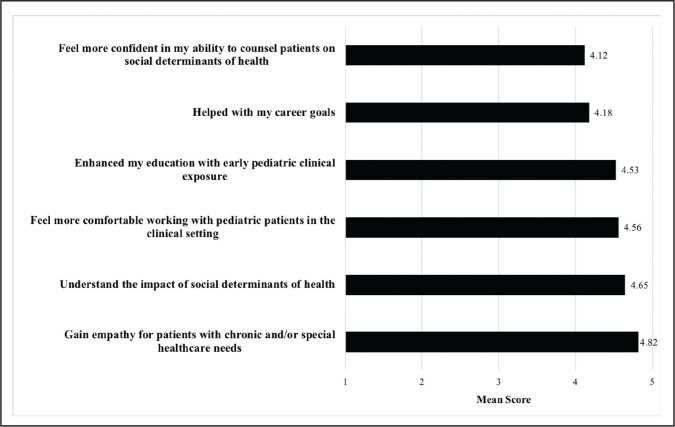
Impact of the Child and Adolescent Motivation and Enrichment Program (CHAMP) participation on medical student mentors. Medical student mentors ranked their agreement with statements numerically from 1 (*strongly disagree*) to 5 (*strongly agree*) based on how participation in CHAMP helped to improve their medical education, empathy, and future career.

Sixteen first-year medical students who served as CHAMP mentors completed surveys after 6 months of CHAMP participation (**Figure 3**). Medical students reported the highest level of agreement that “CHAMP helped me gain empathy for patients with chronic and/or special healthcare needs” (mean = 4.82).

### Nurse Opinions

**Figure 4.**
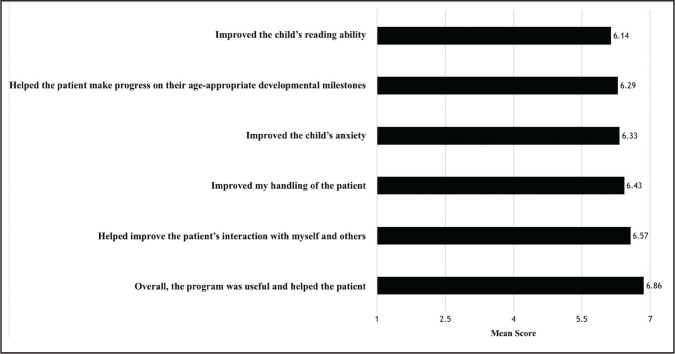
Nurse opinions of developmental gains resulting from Child and Adolescent Motivation and Enrichment Program (CHAMP) participation by hemodialysis patients. Nurses ranked their agreement with statements numerically from 1 (*strongly disagree*) to 7 (*strongly agree*) based on how their patient's participation in CHAMP improved various behavioral, developmental, and psychosocial outcomes.

All seven pediatric dialysis unit nurses completed surveys (**Figure 4**). Nurses responded most highly to “overall, the program was useful and helped the patient” (mean = 6.86). Nurses also reported that CHAMP helped improve the patient's interactions with nursing staff and others (mean = 6.57), and that, from their perspective, patients who participated in CHAMP improved their reading skills (mean = 6.14). In response to the open-ended questions, nurses replied that benefits of CHAMP include “the patients are more interested in learning,” “[the program] helps them with motivation and life counseling,” and “[the program] has been a great support to some of our patients who do not have a lot of support in their lives.” They also remarked that “all patients enjoy their interaction [with their mentors] … they all seem happy and start to cheer when their mentor walks in.” One nurse remarked that the worst part of the program is that mentors are limited in their capacity to help patients, citing an example in which a student mentor wanted to walk a patient to their psychologist appointments to increase compliance and was prohibited from doing so. The best things noted about the program by nurses included the openness of patients to share ideas with mentors, patients looking forward to their mentor meetings, receiving interaction during dialysis to take their mind off of treatment, and seeing the positive response from patients after their mentor interactions.

## Discussion

Given the numerous physical and psychosocial demands of HD, which is further compounded by time spent in the hospital and away from school, it is not surprising that the literacy skills of pediatric and young adult patients who receive dialysis are generally behind those of their grade-level peers. CHAMP was implemented to address a critical need for academic and social enrichment among this unique population. Although the literature pertaining to interventions enhancing quality of life for patients with ESKD is scarce, interventions targeting pediatric patients are especially lacking. Beyond private tutors visiting the dialysis unit during HD sessions, little data exist pertaining to educational support programs for pediatric patients who need dialysis.

Positive disease-related and psychosocial outcomes are evident among the handful of published studies of mentor-ship programs for ESKD patients. St. Clair Russell et al. (2017) described a peer mentorship program during which adult HD patients received weekly mentorship resulting in significant improvement of numerous psychosocial outcomes. Both mentees and mentors, the majority of whom received HD, demonstrated increased knowledge, perceived self-efficacy, and perceived social support (St. Clair Russell et al., 2017). In Japan, the Encourage Autonomous Self-Enrichment Program was adopted in patients with CKD and effectively improved perceived self-efficacy and self-management behaviors in addition to decreasing serum potassium levels (Joboshi & Oka, 2017).

The ultimate goal of CHAMP is that participation will offer improved outcomes for patients on dialysis, which will place their academic and social abilities closer to those of their peers. Initial program surveys suggest that participating patients, medical students, and unit nurses regard the program highly. Patients seem to have garnered increased social support, as they report enjoying time with their mentors and having fun during visits. Increased social support is correlated with improved patient outcomes, reduced hospitalizations, and increased likelihood to complete pre-transplant assessments (Clark et al., 2008; Plantinga et al., 2010). Nurses who observe these interactions during HD sessions additionally feel that the program has benefited their patients and even helped to improve the patient's interactions with others. This could be attributed to improved mood of patients after their mentor visits and, therefore, more positive subsequent interactions. In addition to the important benefits of CHAMP for participating patients, our program provides a unique opportunity for medical students. Pre-clinical experiences through CHAMP bring relevance to the basic science courses that dominate these years. Medical students report increased comfort working with pediatric patients, increased understanding of the social determinants of health, and enhanced empathy for patients with chronic health care needs.

Overall, CHAMP is an innovative program with numerous benefits to both pediatric HD patients and pre-clinical medical students. Although these preliminary proof-of- concept data do not show robust perceived improvements in patients' academic abilities, the psychosocial and motivational aspects of the program are readily apparent to both patients and nurses who observe mentorship interactions. Such a program is critical to HD patients as it assists patients in forming a framework for developing self-esteem and health literacy, which is essential as patients learn to manage their illness as they grow into adulthood. The program design of CHAMP as outlined herein could serve as a template for future medical students to adopt and implement at their institutions so that more children and adolescents with ESKD can benefit.

## Future Directions

Rigorous qualitative and quantitative investigation of the efficacy of our program is warranted to more clearly delineate its academic and psychosocial benefits. Annual assessments of literacy, health literacy, quality of life, and social support will allow for demonstration of patient improvement with time and mentor support. We expect patients who participate in CHAMP to improve their social skills through interactions with their mentor(s), increase their confidence in both social interactions and specific educational capabilities, and increase patient-reported quality of life. Fostering improvements in health literacy and disease-related knowledge could potentially stimulate enhanced awareness and care in patients' dietary choices. Further, we are hopeful that medical student mentor visits will improve the health literacy of participating patients and thereby translate to improved outcomes after kidney transplantation. Designing robust validated tests of motivation, self-efficacy, literacy, and health literacy is important to assess the true efficacy of this program.
